# A systematic review and meta-analysis of sinus laser associated closure (SiLaC) versus endoscopic pilonidal sinus treatment (EPSiT) in the management of pilonidal sinus disease

**DOI:** 10.1007/s00423-026-04026-1

**Published:** 2026-04-11

**Authors:** Zhicheng Li, Lei Jin, Mustapha Ouali, Vincent de Parades, Zhenyi Wang, Jiong Wu, Peter C. Ambe

**Affiliations:** 1 Department of Proctology, Yueyang Hospital of Integrated Traditional Chinese and Western Medicine, Shanghai University of Traditional Chinese Medicine, Shanghai, China; 2 Department of General Surgery and Proctology, University of Sfax, Sfax, Tunisia; 3 Service de Proctologie Médico-Chirurgicale, Hôpital Paris Saint‐Joseph, Paris, France; 4 Faculty of Health, Witten/Herdecke University, Witten, Germany; 5 University of Ghana School of Medicine, Accra, Ghana

**Keywords:** Pilonidal sinus disease, Laser treatment, SiLaC, Endoscopic pilonidal sinus treatment, EPSiT

## Abstract

**Background:**

Various surgical modalities exist for the management of pilonidal sinus disease (PSD), with an increasing trend toward minimally invasive techniques. Sinus Laser associated Closure (SiLaC) and endoscopic pilonidal sinus treatment (EPSiT) are two minimally invasive techniques widely used in clinical practice. However, a systematic comparison of both techniques is lacking. This study conducted separate single-arm analyses of both techniques to compare their efficacy and safety.

**Objective:**

A systematic literature search was performed across electronic databases including Web of Science, Embase, the Cochrane Library, Google Scholar and PubMed to identify relevant studies investigating SiLaC versus EPSiT for PSD. The primary outcome measures included operative time, cure rate, recurrence rate, postoperative complication rate, and total wound healing time.

**Results:**

A total of 29 studies were included. The mean operative time was 18.02 min (95% CI:13.42–22.62; I^2^ = 99.4%; *P* < 0.001) for SiLaC and 30.11 min (95% CI: 21.33–38.88; I² = 98.5%; *P* < 0.001) for EPSiT. The cure rate was 86% (95% CI: 80–91%; I^2^ =92%; *P* < 0.001) in the SiLaC and 88% (95% CI: 83–94%; I² = 87.1%; *P* < 0.001) in the EPSiT group. The pooled recurrence rate was 11% (95% CI: 6–15%; I^2^ = 90.4%; < 0.001) for SiLaC and 9% (95% CI: 5–12%; I² = 82.9%; *P* < 0.001) for EPSiT. The complication rate was 10% (95% CI: 7–14%; I^2^ = 79.6%; *P* < 0.001) for SiLaC and 7% (95% CI: 1–15%; I² = 74.7%; *P* < 0.05) for EPSiT. The mean total healing time was 30.08 days (95% CI: 22.73–37.43%; I^2^ = 99.2%; *P* < 0.001) for SiLaC and 26.55 days (95% CI: 24.40–28.70; I² = 90.9%; *P* < 0.001) for EPSiT.

**Conclusion:**

This indirect comparative analysis suggests comparable efficacy between SiLaC and EPSiT for PSD based on currently available, predominantly single-arm studies. These findings, limited by the lack of direct comparative trials, highlight the urgent need for prospective investigations to establish robust comparative effectiveness.

## Introduction

PSD is a chronic disorder predominantly affecting the sacrococcygeal region, with a higher incidence among young males (approximately 26 per 100,000) [1, 2]. Current evidence identifies obesity, prolonged sedentary behavior, hirsutism, and local perianal irritation as risk factors for PSD. The acute phase is characterized by swelling and pain in the sacrococcygeal area, while the chronic phase often presents with persistent discharge [3].

Although numerous surgical approaches have been described for PSD, ranging from wide excision with midline closure to various flap techniques, yet a standardized surgical protocol remains elusive [4]. An ideal surgical technique should feature minimal tissue less pain, low recurrence rates, short hospitalization, early return to daily activities, minimal scarring, and technical feasibility [5, 6]. Consequently, novel minimally invasive techniques have gained considerable traction in clinical practice [4].

Among minimally invasive interventions, laser therapy and endoscopic treatment are currently widely used. Endoscopic treatment for PSD was first described by Meinero in 2013, and its core principle involves the visual debridement of sinus tract contents and hair debris using a fistuloscope system [5]. In contrast, laser ablation (or laser-assisted closure) for PSD employs a laser fiber to thermally ablate and obliterate the sinus tract [7]. Existing literature has validated high clinical success rates with acceptable recurrence rates for both techniques [8, 9]. Recently, an international consortium of experts from the International Society of Laser Proctology, ISoLP published a position paper specifically addressing the standardization of SiLaC and its combined application with EPSiT (E-SiLaC) [10]. This position paper outlines indications, procedural details, and attempts to homogenize practice for both stand-alone laser and combined laser-endoscopic approaches. Importantly, the authors of this position paper explicitly acknowledge the foundational limitation of the current evidence base, stating that it is “limited to small retrospective series“ [10]. Therefore, the present study aimed to synthesize available evidence via a systematic review and meta-analysis to evaluate and compare the efficacy and safety of laser therapy versus endoscopic treatment in the management of PSD.

## Methods

This study was conducted in accordance with the Cochrane Handbook Systematic Reviews of Interventions and reported following the PRISMA (Preferred Reporting Items for Systematic Reviews and Meta-Analyses) guidelines. A systematic literature search was performed using the Web of Science, Embase, Cochrane Library, PubMed and ClinicalTrials.gov. Search terms included: “PSD,” “pilonidal sinus,” “pilonidal sinus disease,” “pilonidal disease,” “SiLaC,” “SILAT,” “Laser,” “Laser Ablation,” “EPSiT,” “ClinicalTrials.gov,”and “endoscopic pilonidal sinus treatment.” The systematic review protocol was registered on PROSPERO(CRD420251274562).

### Inclusion and exclusion criteria (PICOS)

Given the scarcity of direct comparative studies between SiLaC and EPSiT, separate single-arm analyses were performed. *For the sake of uniformity*,* SiLaC was used as an umbrella term for all laser – assisted procedures for PSD (SiLaC*,* SiLaT*,* PiLaT*,* etc.)*.

- **P (Population)**: Patients diagnosed with pilonidal sinus disease.
- **I (Intervention/Comparator)**: Patients undergoing laser ablation or endoscopic treatment.
- **(Outcomes)**: Studies reporting any of the following outcomes: operative time, cure rate, recurrence rate, postoperative complications, and total healing time.
- **S (Study Design)**: Randomized or non-randomized clinical trials, observational studies (cohort or case-control).

Exclusion criteria were as follows: Irrelevant articles, editorials, letters, case reports, reviews, and meta-analyses. Studies with fewer than three patients or those combining the investigated techniques with other surgical methods were also excluded. Articles containing duplicate data or not explicitly reporting the primary outcomes of this review were excluded.

### Study selection and data extraction

Two reviewers (ZL and LJ) independently performed the selection and extraction processes. Disagreements were resolved with the senior investigators (JW and PCA). The first step involved screening titles and abstracts by both researchers to identify potentially eligible articles. Furthermore, the reference lists of eligible studies were manually searched for additional potential articles. Duplicate records were removed. Finally, full texts were independently assessed for eligibility by the two researchers, with inconsistencies resolved through discussion with PCA and JW.

### Data analysis and synthesis

Statistical analysis was performed using Stata software. For dichotomous variables (e.g., healing rate, recurrence rate), risk ratios (RR) and 95% confidence intervals (CI) were calculated. For continuous data, the chi-square test and I² statistic were used to assess heterogeneity and determine the consistency of results across different studies.

## Results

### Search results

**Fig. 1:**
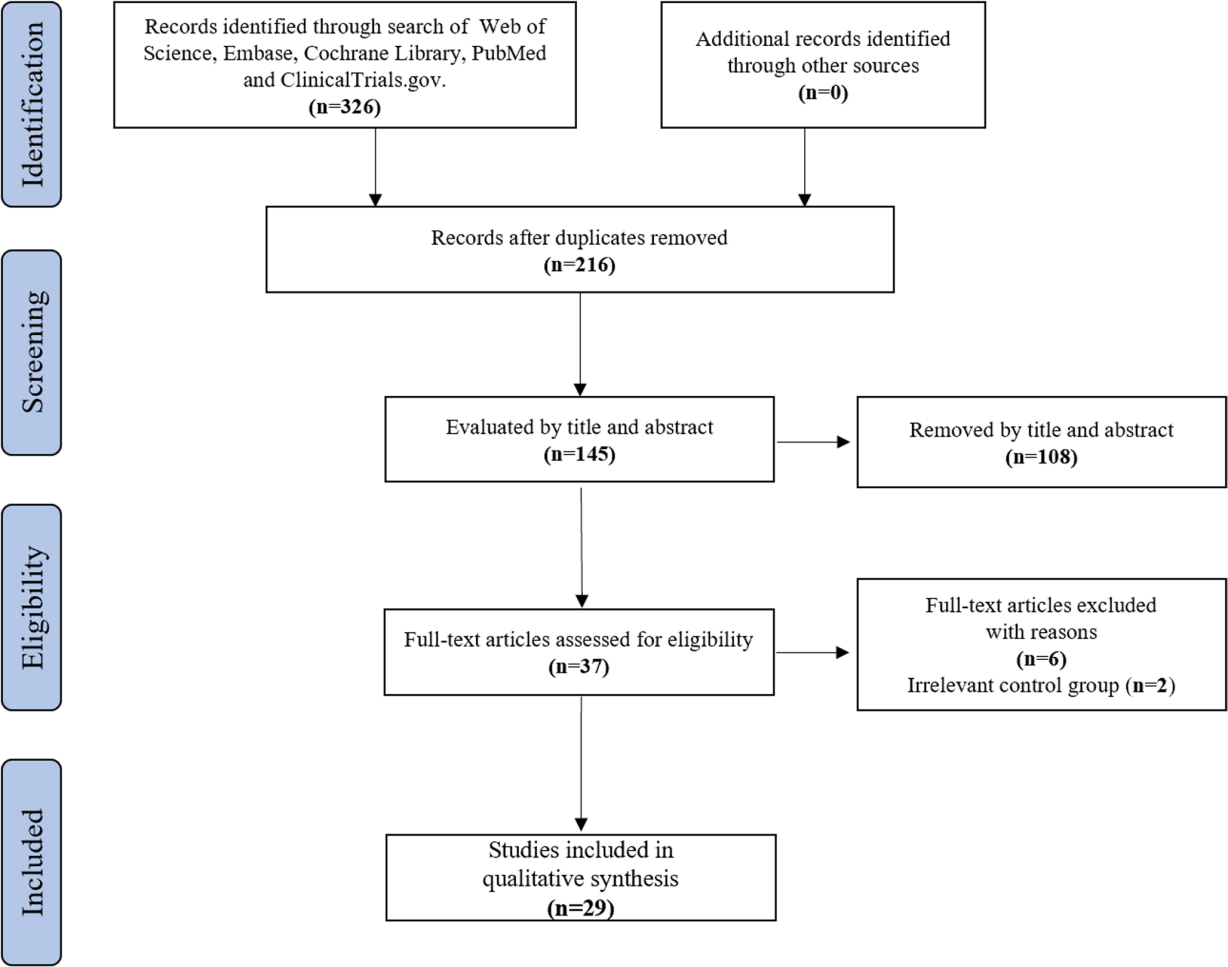
Flow chart of the systematic review

A total of 326 articles were retrieved from the databases. After removing duplicates and screening titles and abstracts, 29 studies met the inclusion criteria and were finally included in this meta-analysis [6, 7, 11–37]. The detailed literature screening process is shown in Fig. 1.

### Characteristics of the included studies

Overall, this review included 19 studies on SiLaC and 10 studies on EPSiT. The basic characteristics of these studies are presented in Table 1.

### Risk of bias evaluation

The methodological quality of the studies was assessed independently by two authors (ZL and LJ) using the National Institute for Health and Care Excellence (NICE) quality assessment checklist for case series. Any discrepancies in interpretation were resolved through discussion with the third author (JW). Study quality was categorized as good (score = 7–8), moderate (score = 4–6), or poor (score = 0–3). The results are summarized in Table 2.

### Operation time

**Fig. 2:**
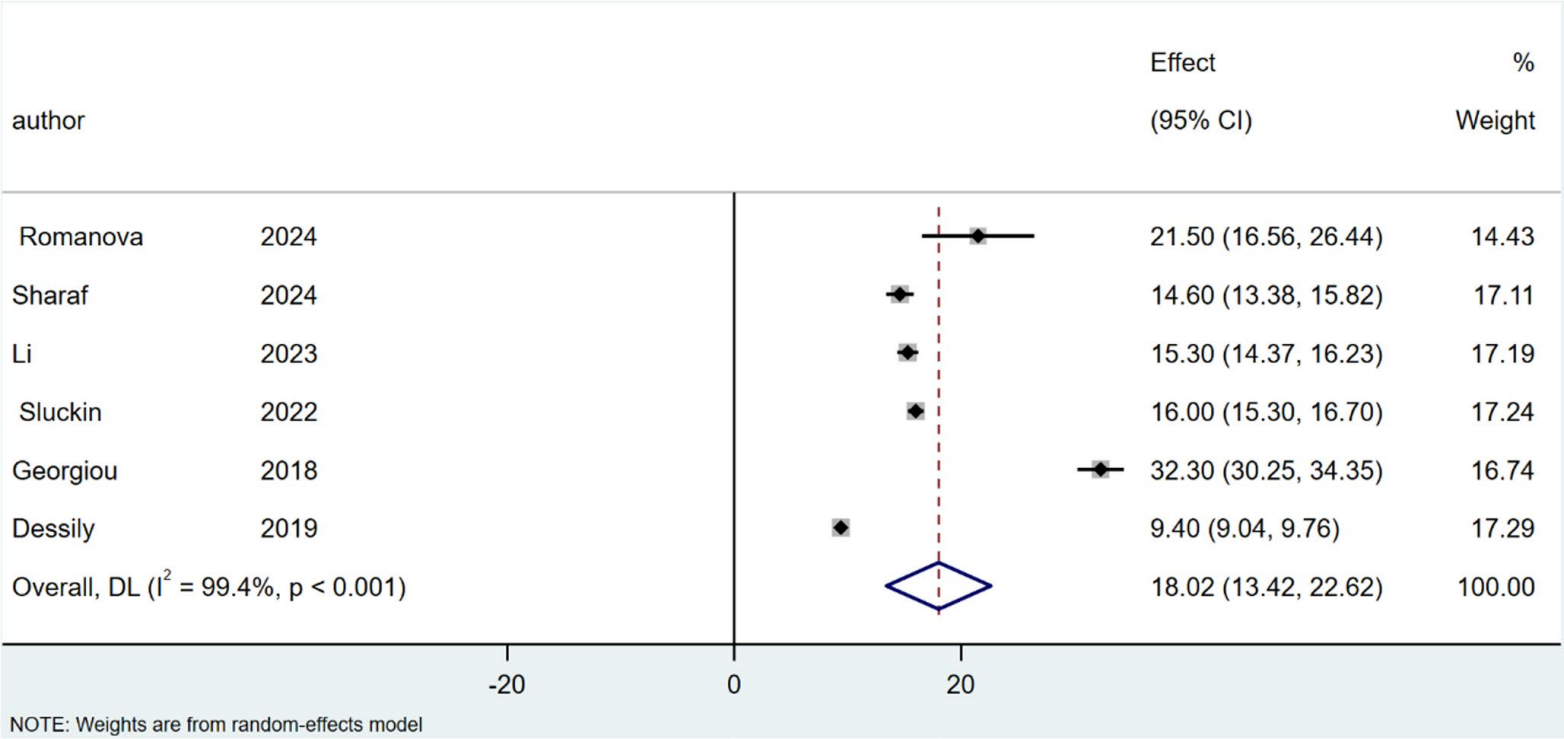
Forest plots of operation time in laser therapy group

**Fig. 3:**
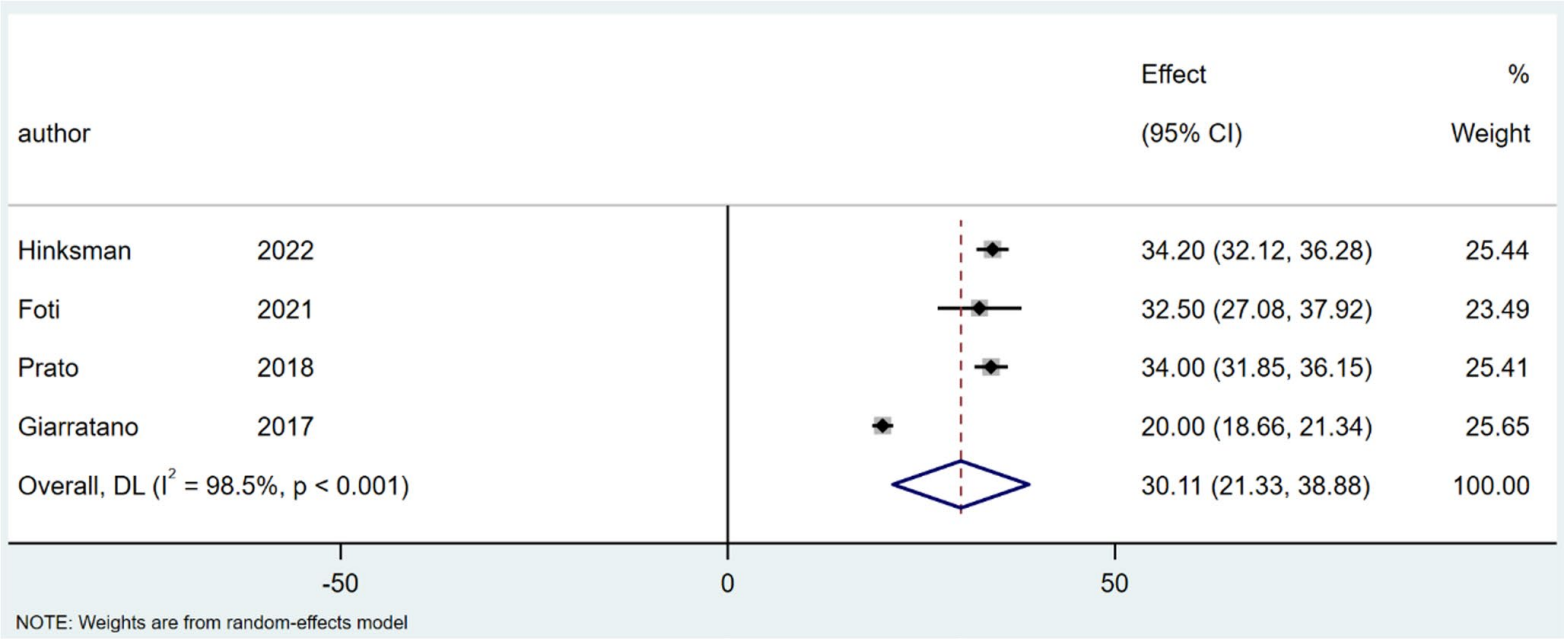
Forest plots of time in endoscopic treatment group

Regarding operation time, the mean lengths of surgery for SiLaC was 18.02 min (95% CI: 13.42–22.62; I^2^ = 99.4%; *P* < 0.001), Fig. 2, and 30.11 min for EPSiT (95% CI: 21.33–38.88; I² = 98.5%; *P* < 0.001), Fig. 3.

### Cure rate

**Fig. 4:**
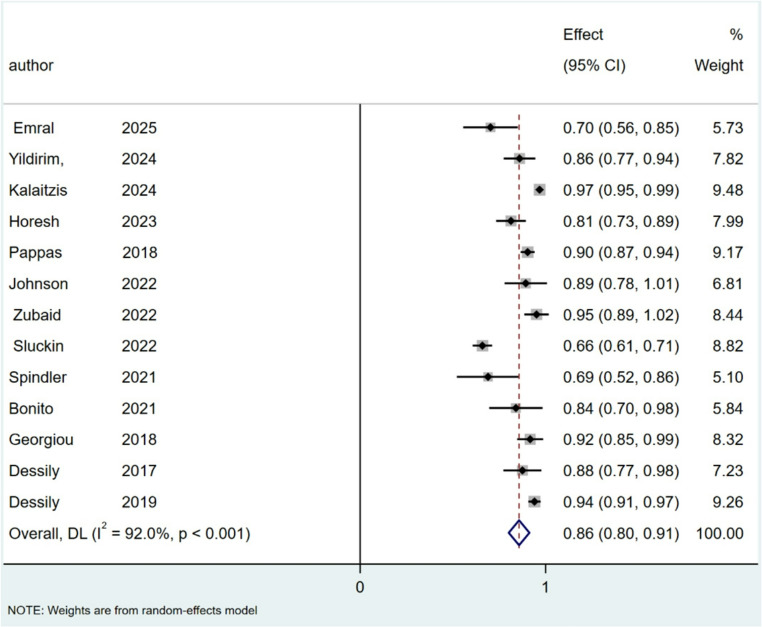
Forest plots of cure rate for SiLaC

**Fig. 5:**
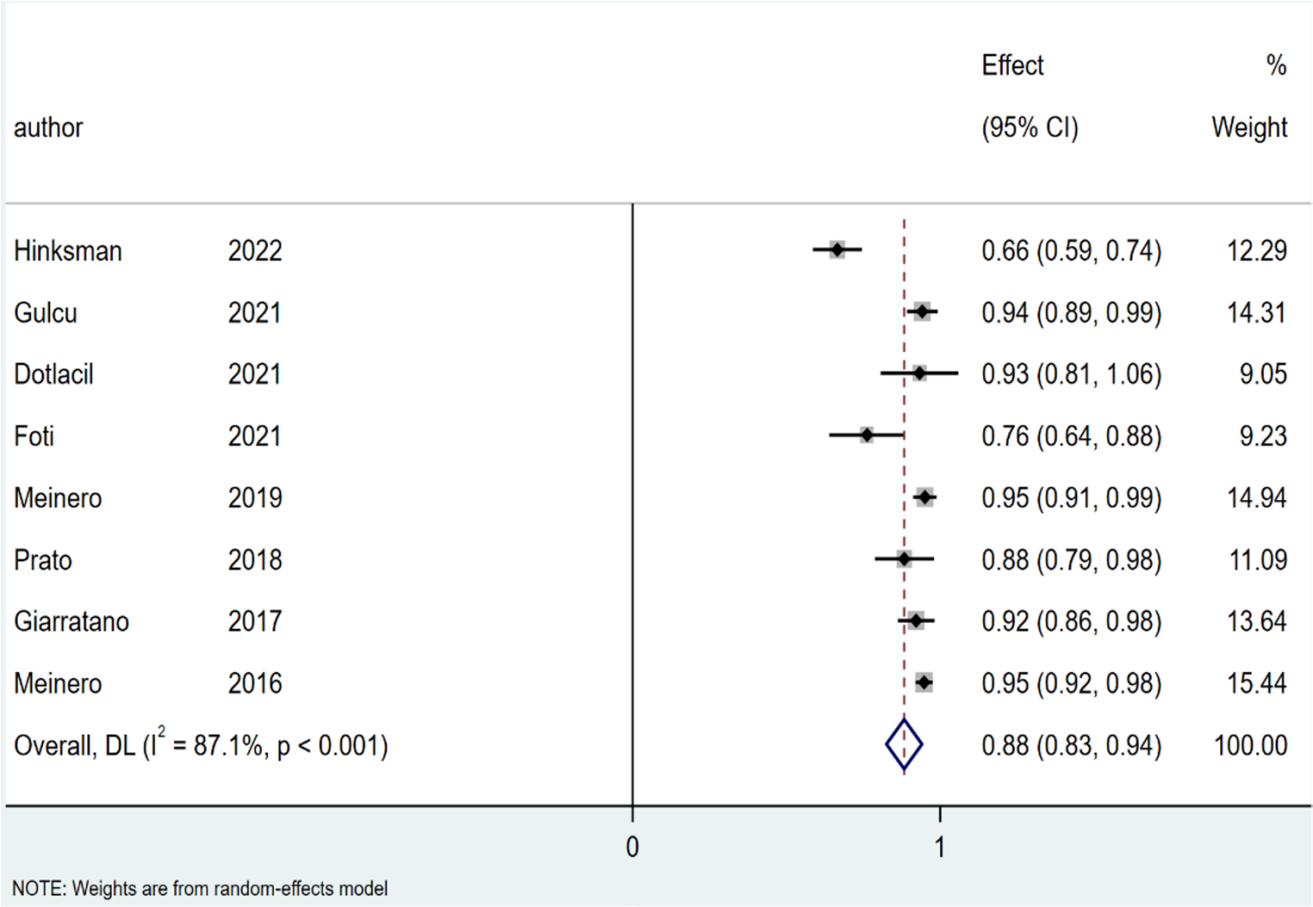
Forest plots of cure rate in endoscopic treatment group

The pooled cure rate of SiLaC was 86% (95% CI: 80–91%; I^2^ = 92%; *P* < 0.001), Fig. 4. For EPSiT the pooled cure rate was 88% (95% CI: 83–94%; I² = 87.1%; *p* < 0.001), Fig. 5.

### Recurrence rate

**Fig. 6:**
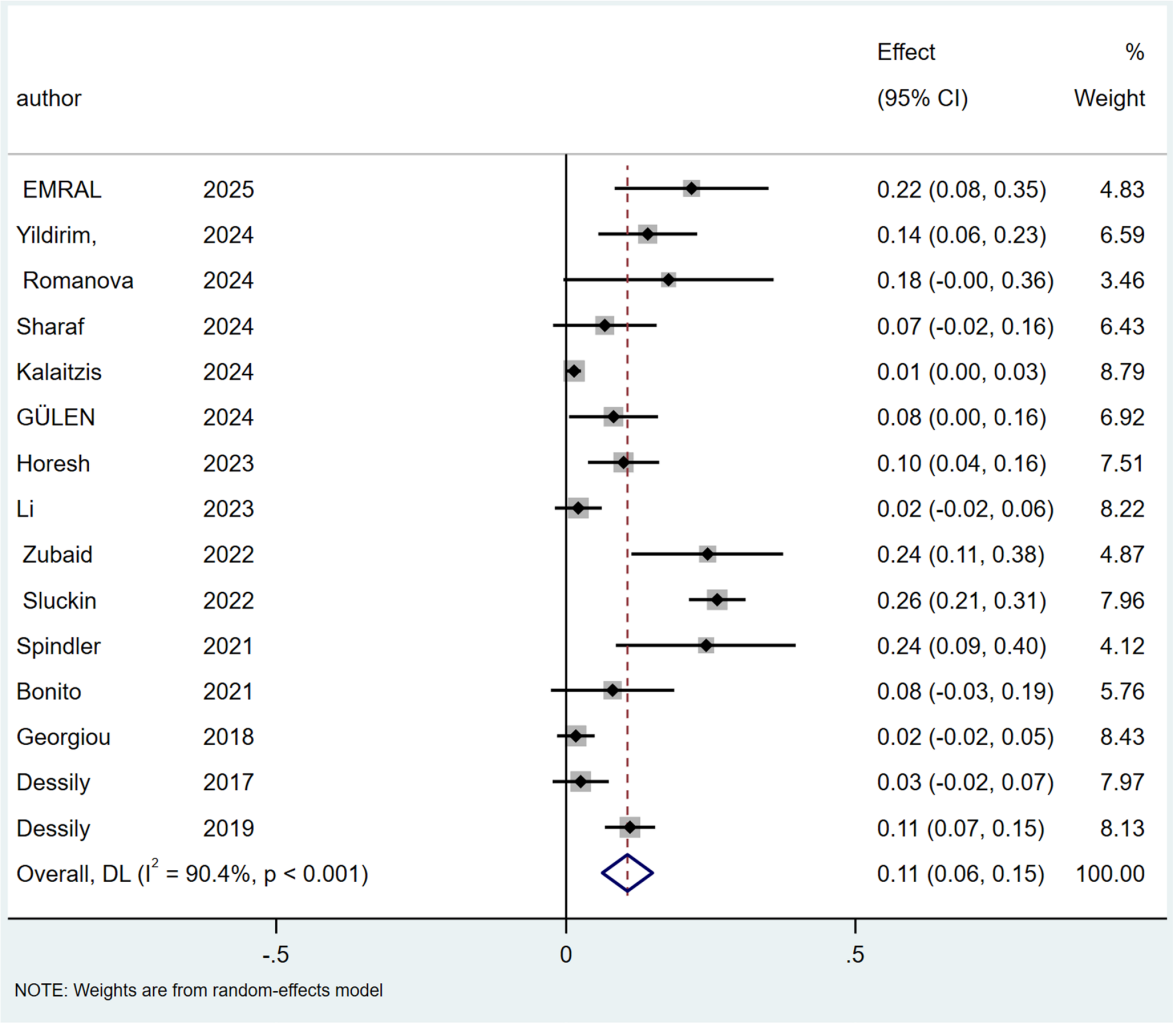
Forest plots of recurrence rate for SiLaC

**Fig. 7:**
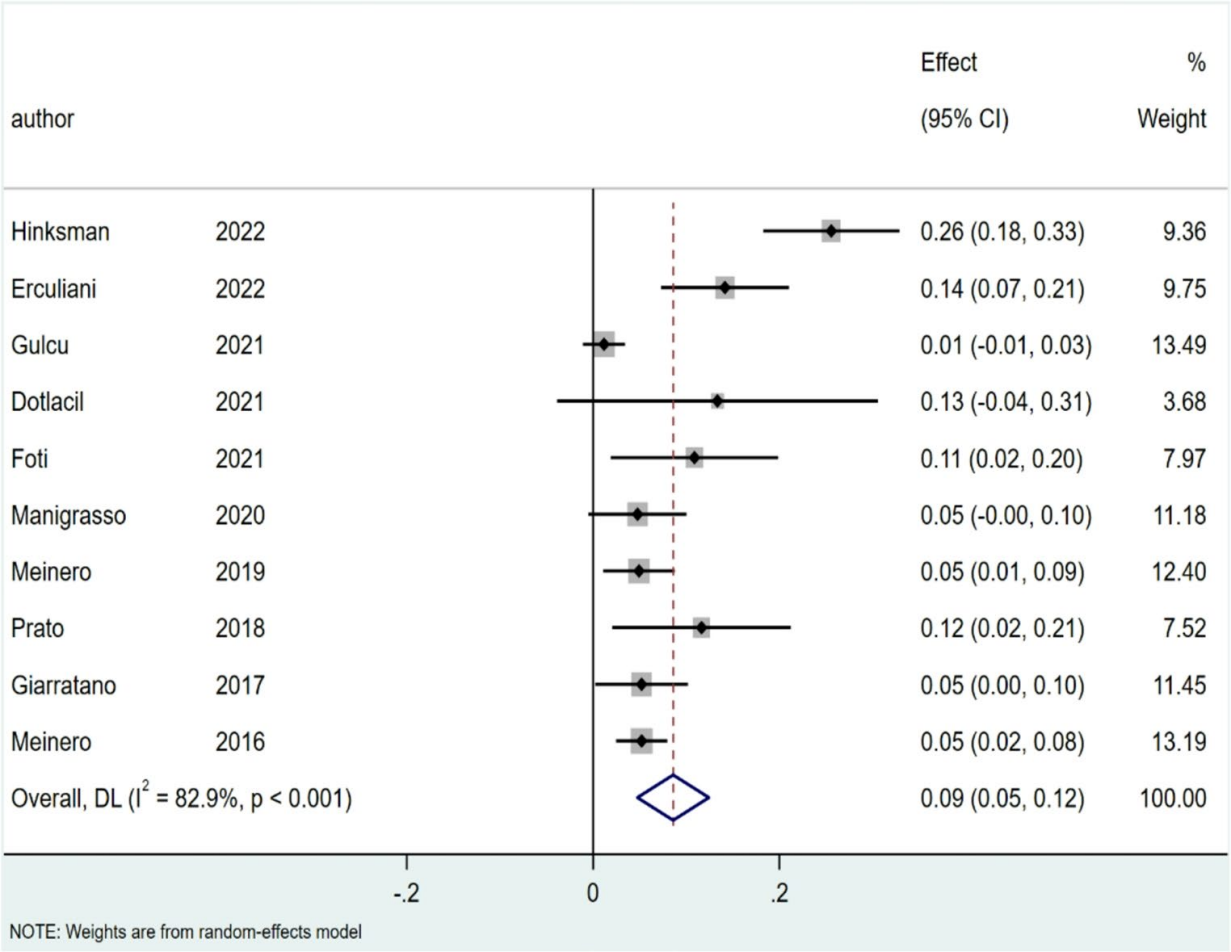
Forest plots of recurrence rate for EPSiT

The rate of recurrence was 11% for SiLaC (95% CI: 6–15%; I² = 90.4%; *p* < 0.001), Fig. 6 and 9% for EPSiT (95% CI: 5–12%; I² = 82.9%; *P* < 0.001), Fig. 7.

### Complication rate

**Fig. 8:**
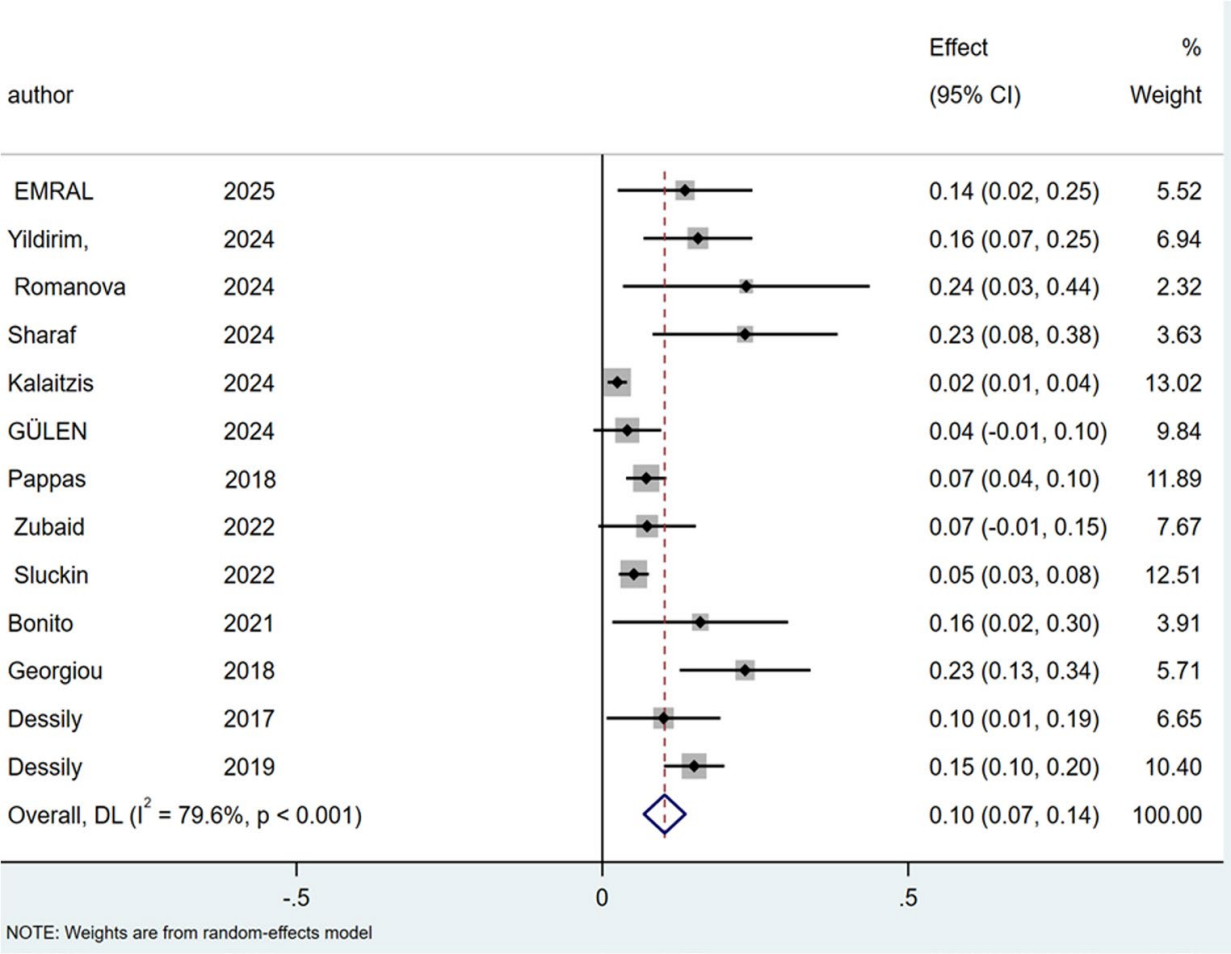
Forest plots of complication rate in laser therapy group

**Fig. 9:**
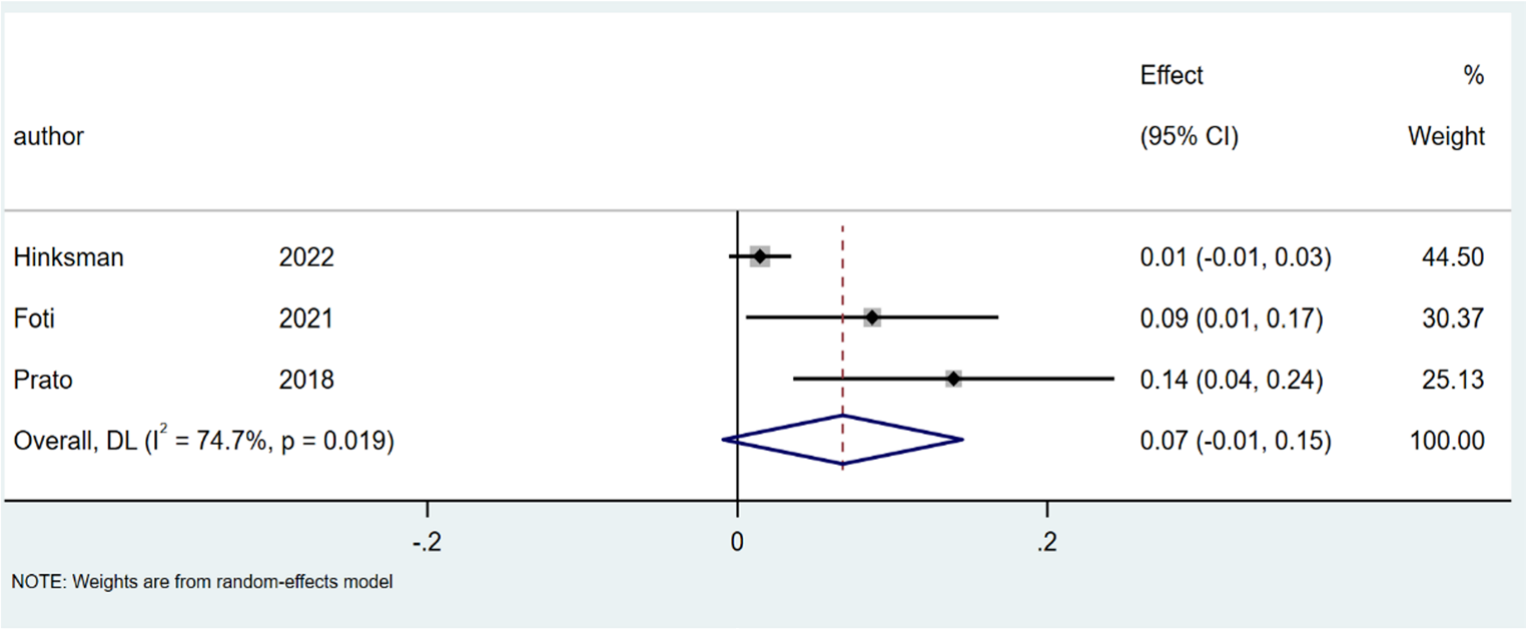
Forest plots of complication rate in endoscopic treatment group

The complication rate was 9% for SiLaC (95% CI: 6–12%; I² = 74.6%; *p* < 0.001), Fig. 8 and 7% for EPSiT (95% CI: 1–15%; I² = 74.7%; *P* < 0.05), Fig. 9.

### Healing time

**Fig. 10:**
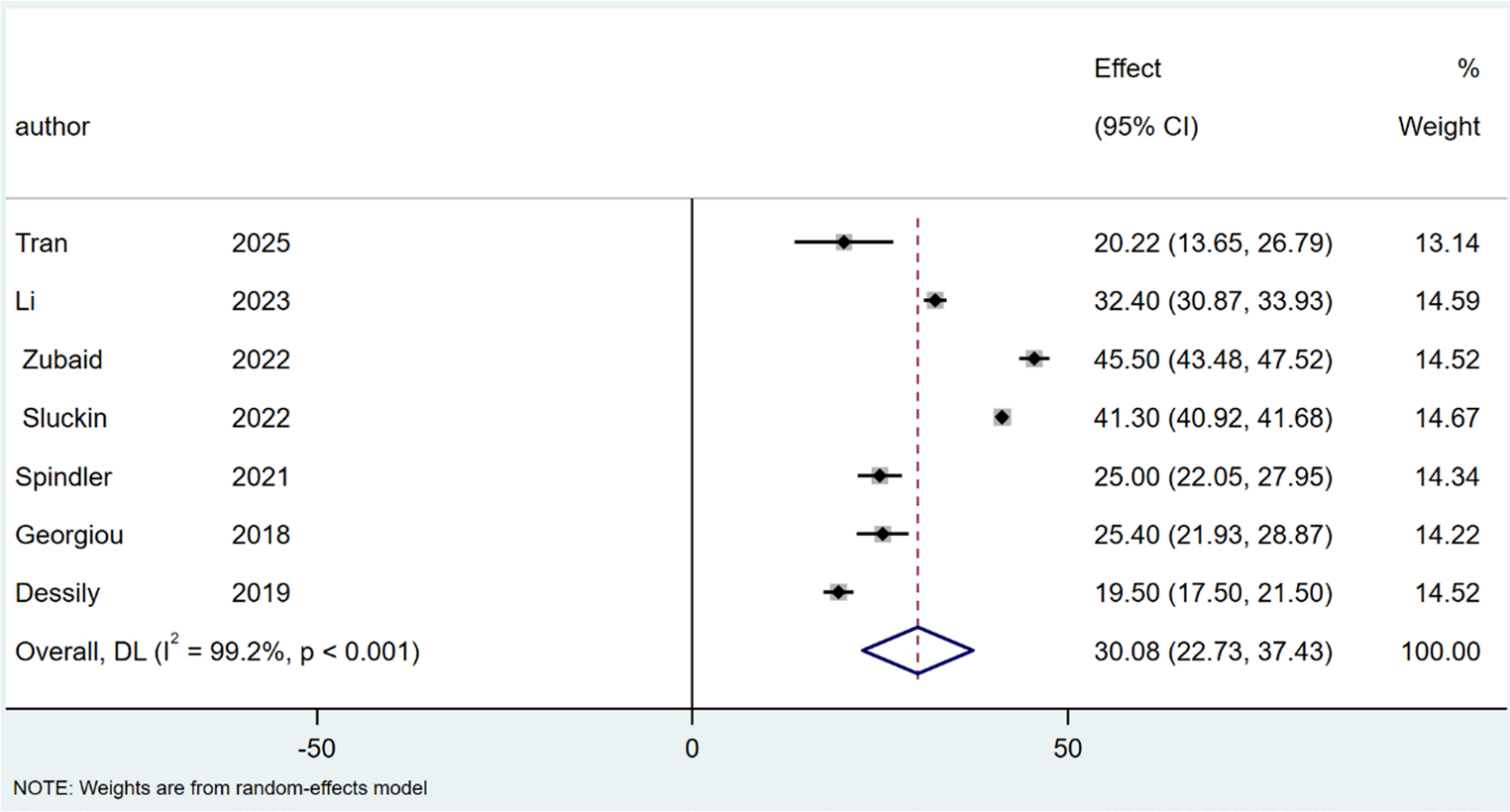
Forest plots of healing time in laser therapy group

**Fig. 11:**
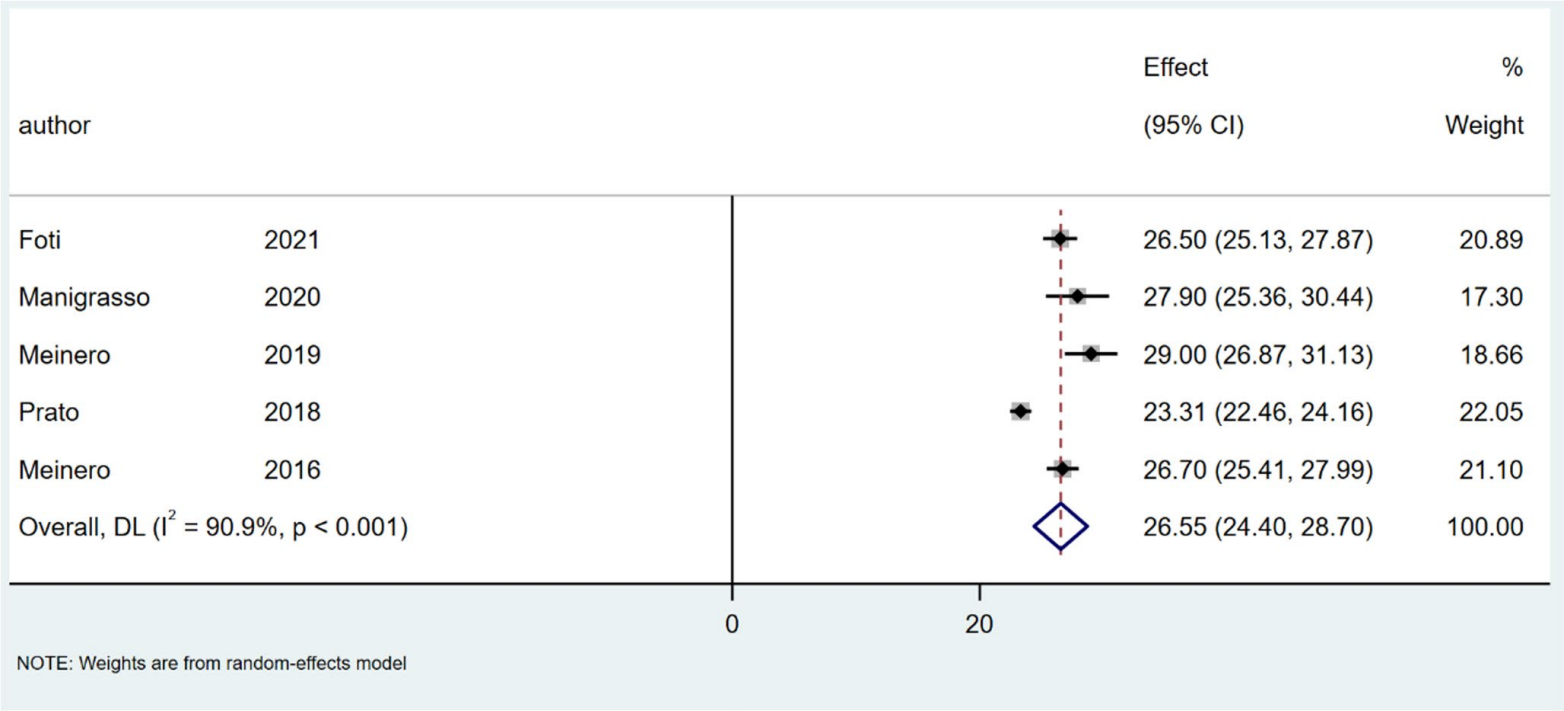
Forest plots of healing time in endoscopic treatment group

The mean time to complete healing was 30.08 days (95% CI: 25.86–38.19; I² = 98.6%; *P* < 0.001) after SiLaC, Fig. 10 and 26.55 days (95% CI: 24.40–28.70; I² = 90.9%; *P* < 0.001) after EPSiT, Fig. 11.

## Discussion

Both laser therapy and endoscopic treatment are well-established minimally invasive surgical modalities for pilonidal sinus disease in recent years [11, 12, 25, 37, 38]. Although both procedures are being increasingly adopted for the management of patients with PSD little or no data on comparisons of both interventions exist in the current literature. The aim of this systematic review and meta-analysis was to fill this gap in the literature. The current literature is void of publications with direct comparisons between SiLaC and EPSiT. Thus, meta-analysis with direct comparison of both interventions could not be performed.

A cohort study by Ersavas et al. involving 73 patients undergoing either SiLaC or EPSiT demonstrated no statistically significant differences in terms of recurrence rate, operative time, complication rate, wound healing time, and time to return to normal daily activities [20], largely supporting the results reported in this study.

Although direct comparison of the results of the two procedures is not feasible due to lack of studies directly comparing both procedures, the pooled results indicated that laser therapy and endoscopic treatment yielded comparable cure rates, recurrence rates and complication rates. The duration of surgery represents the only difference identified amongst both interventions. Although SiLaC was relatively faster compared to EPSiT, the confidence interval (CI) of the SiLaC group was overall much lower than the estimated value of the EPSiT group. However, the CIs of the two interventions slightly overlap at the edge. This finding suggested that while SiLaC may be a slightly faster procedure, EPSiT may have a similarly short duration of surgery in very experienced hand.

While a visualized exploration of the sinus tract to avoid missing branches and the possibility of a complete destruction of granulation tissue and radical debridement under visual guidance have been thought as advantages of EPSiT, these argumentations remain debatable from a clinical standpoint. First, these aspects inevitable prolong the duration of surgery. Second, the endoscopic procedure requires not only investing in specialized equipment, but also demand for additional training. Therefore, the endoscopic procedure is associated with a higher treatment cost compared to the SiLaC alone [25]. It is therefore questionable whether or not the cost incurred by the endoscopic technique is justified by the 4 days difference in complete wound healing, as this seems to be the only positive difference between SiLaC and EPSiT. This critical thinking is back by the results from the study by Bilgin et al. reporting on 106 patients undergoing minimally invasive treatment for PSD, including 73 cases with a combination of Laser and EPSiT. The study failed to show any significant benefits in postoperative outcomes, including no obvious improvements in pain, recovery time, complication rate, readmission rate, recurrence rate and patient satisfaction [39]. Contrary to the data published by Bilgin et al., Mustapha Ouali reported a 95% rate of complete epithelialization within 3 weeks and 3.3% recurrence rate at 6 month using the combination of SiLaC and EPSiT [10]. A similar finding was reported by Gulcu et al. for wound healing following combined Laser and EPSiT [25]. Although the literature on this topic is not conclusive and hardly any consistent patterns were observed, experts from the International Society of Laser Proctology (ISoLP) acknowledge some advantages of combining SiLaC with EPSiT, which has been termed E-SiLaC. In their recently published position paper on SiLaC and EPSiT, ISoLP suggests an individualized assessment of patients to identify candidates who may benefit from E-SiLaC [10].

### Limitations

Some relevant limitations to this study need to be discussed. First, direct comparison between both interventions was not possible due to lack of studies. Thus, a single intervention meta-analysis was performed for each treatment arm. Second, all studies included in this systematic review are of retrospective design with well - known limitations, small sample sizes, short follow-up durations and heterogeneity of available data limit the precision and interpretability of the pooled estimates reported in this work. Third, variability in surgical techniques across different centers with subsequent heterogeneity in outcomes may have influenced the overall results seen in this study. Forth, there is a possibility that some potentially eligible studies were not identified using our search strategy. Finally, a formal cost-effectiveness analysis was beyond the scope of this review, but the economic implications of equipment, operative duration, and time to return to work constitute an important area for future comparative research.

## Conclusion

Based on current evidence, SiLaC and EPSiT are effective minimally invasive treatments for PSD, with comparable healing and recurrence rates. The selection of surgical modality should be individualized, comprehensively considering equipment availability, surgeon proficiency, and patient preference.

**Table 1:** Characteristics of the included studies

Author year	Type of article	Period		Country	*n*	Treatment	Main outcomes
Tran,2025	retrospective	December 2024 to July 2025	PSD	Vietnam	9	SiLaC	Operative time, complications rate, healing time, healing rate, recurrence rate
EMRAL, 2025	retrospective	January 2022 and January 2025	recurrent PD	Turkey	37	SiLaC	Healing rate, recurrence rate, complications rate
Romanova,2024	retrospective	2019 to 2023	PD	Germany	17	SiLaC	Recurrence rate, complications rate, operative time
Yildirim,2024	retrospective	March 2020 to December 2023	PD	Turkey	64	SiLaC	Recurrence rate, complications rate, healing rate
Sharaf,2024	retrospective	March 2022 to June 2022	PD	Egypt	30	SiLaC	Recurrence rate, complications rate
Kalaitzis,2024	retrospective	January 2018 to January 2024	PD	-	368	LaPSe	Healing rate, recurrence rate, complications rate
Al-Khazraji,2024	prospective	February 2023 and February 2024	PSD	Iraq	50	SiLaT	Complications rate
GÜLEN,2024	retrospective	August 2020 and August 2023	PSD	Turkey	49	SiLaC	Complications rate, recurrence rate
Horesh,2023	retrospective	September 2018 and December 2020	PSD	Israel	92	SiLaC	Healing rate, recurrence rate
Pappas,2018	prospective	June 2012 to December 2015	PSD	Greece	237	SiLaT	Healing rate, Complications rate
Li,2023	retrospective	March 2019 and July 2022	SPD	China	48	Silac	Healing rate, recurrence rate, operative time, healing time
Johnson,2022	prospective	April 2016 and July 2019	PSD	UK	35	PiLAC	Healing rate, recurrence rate,
Hinksman,2023	Retrospective	January 2014 to November 2019	PSD	Australia	137	EPSIT	Return to work, Return to work, pain
Erculiani,2022	retrospective	January 2017 and December 2021	PSD	Italy	115	EPSiT	Recurrence rate
Zubaid,2022	retrospective	February 2012 to December 2019	PSD	SAU	41	SiLaT	Complications rate, healing time, recurrence rate
Sluckin,2022	cohort	January 2017 to March 2020	PSD	The Netherlands	311	SiLac	Recurrence rate, complications rate
Foti,2021	retrospective	March 2015 to December 2019	PSD	Italy	42	EPSiT	Operative time, complications rate, healing time, recurrence rate, healing rate
Bonito,2021	retrospective	July 2018 to August 2020	PSD	Portugal	27	SiLac	Healing rate, recurrence rate, complications
Spindler,2021	retrospective	June 2018 to August 23 2019	PSD	France	29	SiLac	Healing rate, healing time, complications rate
Dotlacil,2021	retrospective	November 2018 to February 2020	PSD	Czech	17	EPSiT	Complications rate, recurrence rate
Gulcu,2021	prospective	May 2018 to December 2019	PSD	Turkey	86	EPSIT	Operative time, complications rate, healing time
Manigrasso,2020	Retrospective	January 2014 to December 2018	recurrent PSD	Italy	63	VAAPS	Recurrence rate, healing time
Dessily,2019	Retrospective	March 2015 to August 2017	PSD	Belgium	200	SiLac	Healing rate, healing time, operative time, recurrence rate, complications rate
Meinero,2019	prospective	March 2012 to December 2014	recurrent PSD	Italy	122	EPSIT	Healing rate, healing time,
Georgiou,2018	prospective	April 2015 and December 2016	PSD	Greece	60	PiLaT	Satisfaction, success rate, VAS pain scores
Prato,2018	prospective	July 2015 and March 2017	PSD	Italy	43	EPSiT	Recurrence rate, complications rate, healing rate
Dessily,2017	retrospective	September 2014 and September 2015	PSD	Belgium	40	SiLac	Healing rate, recurrence rate, complications rate
Giarratano,2017	prospective	October 2013 through November 2015	PSD	Italy	77	EPSiT	Operative time, healing rate
Meinero,2016	prospective	March 2012 to December 2014	PSD	Switzerland,	250	EPSiT	Healing rate, healing time, recurrence rate

**Table 2:** Assessment of the methodological quality of studies included in the review

Study	Multicenter study	Clearly defined objective	Reported inclusion and exclusion criteria	Clearly defined outcomes	Prospective data collection	Patients recruited consecutively	Clearly described results of the study	Stratified outcomes	Total score
Tran,2025	0	1	1	1	0	1	1	1	6
EMRAL, 2025	0	1	1	1	0	1	1	1	6
Romanova,2024	0	1	1	1	0	1	1	1	6
Yildirim,2024	0	1	1	1	0	1	1	1	6
Sharaf,2024	0	1	1	1	0	1	1	1	6
Kalaitzis,2024	0	1	1	1	0	1	1	1	6
Al-hazraji,2024	0	1	1	1	1	1	1	1	7
GÜLEN,2024	0	1	1	1	0	1	1	1	6
Horesh,2023	0	1	1	1	0	1	1	1	6
Pappas,2023	0	1	1	1	1	1	1	1	7
Li,2023	0	1	1	1	0	1	1	1	6
Johnson,2022	0	1	1	1	1	1	1	1	7
Hinksman,2023	0	1	1	1	0	1	1	1	6
Erculiani,2022	0	1	1	1	0	1	1	1	6
Zubaid,2022	0	1	1	1	0	1	1	1	6
Sluckin,2022	0	1	1	1	0	1	1	1	6
Foti,2021	0	1	1	1	0	1	1	1	6
Bonito,2021	0	1	1	1	0	1	1	1	6
Spindler,2021	0	1	1	1	0	1	1	1	6
Dotlacil,2021	0	1	1	1	0	1	1	1	6
Gulcu,2021	0	1	1	1	1	1	1	1	7
Manigrasso,2020	0	1	1	1	0	1	1	1	6
Dessily,2019	0	1	1	1	0	1	1	1	6
Meinero,2019	0	1	1	1	1	1	1	1	7
Georgiou,2018	0	1	1	1	1	1	1	1	7
Prato,2018	1	1	1	1	1	1	1	1	8
Dessily,2017	0	1	1	1	0	1	1	1	6
Giarratano,2017	0	1	1	1	1	1	1	1	7
Meinero,2016	1	1	1	1	1	1	1	1	8

## Data Availability

All data besides what is published can be requested via the corresponding authors.

## References

[1] Harries RL, Alqallaf A, Torkington J et al (2019) Management of sacrococcygeal pilonidal sinus disease[J]. Int wound j 16(2):370–378 30440104 10.1111/iwj.13042 PMC7949345

[2] Søndenaa K, Andersen E, Nesvik I et al (1995) Patient characteristics and symptoms in chronic pilonidal sinus disease[J]. Int j colorectal dis 10(1):39–42 7745322 10.1007/BF00337585

[3] Al-Homoud SJ, Habib ZS, Abdul Jabbar AS et al (2001) Management of sacrococcygeal pilonidal disease[J]. Saudi med j 22(9):762–764 11590447

[4] Wiinblad IM, Ulrichsen J, Brandstrup B (2025) Outcome After Surgical Treatment for Chronic Pilonidal Sinus Disease: A Systematic Review of Common Surgical Techniques[J]. Dis Colon Rectum 68(5):515–529 39982788 10.1097/DCR.0000000000003688

[5] Meinero P, Mori L, Gasloli G (2014) Endoscopic pilonidal sinus treatment (E.P.Si.T.)[J]. Tech coloproctol 18(4):389–392 23681300 10.1007/s10151-013-1016-9

[6] Li Z, Jin L, Gong T et al (2023) An effective and considerable treatment of pilonidal sinus disease by laser ablation[J]. Laser med sci 38(1):82 10.1007/s10103-023-03741-1 PMC9977879 36856904

[7] Dessily M, Dziubeck M, Chahidi E et al (2019) The SiLaC procedure for pilonidal sinus disease: long-term outcomes of a single institution prospective study[J]. Tech coloproctol 23(12):1133–1140 31773347 10.1007/s10151-019-02119-2

[8] Manigrasso M, Anoldo P, Cantore G et al (2021) Endoscopic Treatment of Pilonidal Sinus Disease: State of Art and Review of the Literature[J]. Front Surg 8(–):812128 35059431 10.3389/fsurg.2021.812128 PMC8764178

[9] Romic I, Augustin G, Bogdanic B et al (2022) Laser treatment of pilonidal disease: a systematic review[J]. Lasers Med Sci 37(2):723–732 34291332 10.1007/s10103-021-03379-x

[10] Ouali M (2025) SiLaC^®^ with EPSiT^®^: early outcomes of laser-endoscopic therapy for pilonidal sinus[J]. Front Surg 12(–):1587467 40352310 10.3389/fsurg.2025.1587467 PMC12062001

[11] Emral AC, Yazici SE (2025) Evaluation of laser ablation for recurrent pilonidal sinus disease: treatment success, recurrence rates, and patient outcomes[J]. Lasers Med Sci 40(1):281 40522552 10.1007/s10103-025-04525-5 PMC12170725

[12] Dung Ngoc T, Dung Quang L, Anh Tu N et al (2025) Sinus laser-assisted closure – SiLaC: Early outcomes and influencing factors[J]. GSC Adv Res Rev 24(2):295–301

[13] Yildirim M, Koca B (2024) Results of Laser-assisted Closure (SiLaC) Surgery in Pilonidal Sinus Disease: Factors Associated With Success[J]. Surg Laparosc Endosc Percutan Tech 34(5):524–528 39140695 10.1097/SLE.0000000000001316

[14] Romanova A, Nissen M, Alrefai M et al (2024) Adolescent pilonidal disease laser treatment (a-PiLaT): a pilot study[J]. Tech Coloproctol 28(1):104 39141158 10.1007/s10151-024-02972-w PMC11324676

[15] Kalaitzis J (2024) A new laser-assisted pilonidal sinus excision surgical technique. presentation of first 368 cases: short- and mid-term results[J]. Med Res Arch 12(6)

[16] Ali Fouad Rashid A-K (2024) Treatment of Pilonidal Sinus Using 1470nm Diode Laser as Minimally Invasive Technique[J]. Ijl 23(2):131–144

[17] Sharaf M, Muhammad I, Mansour B (2023) Evaluation of laser ablation in treatment of pilonidal sinus short title: laser in pilonidal sinus[J]. Al-Azhar Int Med J 4(12)

[18] Johnson DE, Granville R, Lovett E et al (2023) Pilonidal sinus laser-assisted closure (PiLAC) - a low-morbidity alternative to excision with excellent long-term outcomes[J]. Ann roy coll surg 105(2):132–135 10.1308/rcsann.2022.0005 PMC9889171 35446708

[19] Horesh N, Meiri H, Anteby R et al (2023) Outcomes of Laser-Assisted Closure (SiLaC) Surgery for Chronic Pilonidal Sinus Disease[J]. J laparoendosc adv s 33(6):556–560 10.1089/lap.2022.0567 36888964

[20] Ersavas C, Erginel B, Yanar F et al (2023) Endoscopic pilonidal sinus treatment (EPSIT) versus sinus laser therapy (SiLaT) for sacrococcygeal pilonidal sinus[J]. Videosurgery miniinv 18(1):144–148 10.5114/wiitm.2022.124206 PMC10091928 37064557

[21] Zubaidi AM, Alali MN, Alshammari SA et al (2022) Outcomes of Sinus Laser Therapy in Sacrococcygeal Pilonidal Sinus Disease: A Single-Center Experience[J]. Cureus 14(9):e29388 36304355 10.7759/cureus.29388 PMC9586186

[22] Spindler L, Alam A, Fathallah N et al (2022) Extensive suppuration and being overweight are factors associated with the failure of laser treatment for pilonidal disease: lessons from the first French retrospective cohort[J]. Tech coloproctol 26(2):143–146 34855026 10.1007/s10151-021-02552-2

[23] Sluckin TC, Hazen SJA, Smeenk RM et al (2022) Sinus laser-assisted closure (SiLaC^®^) for pilonidal disease: results of a multicentre cohort study[J]. Tech coloproctol 26(2):135–141 34993686 10.1007/s10151-021-02550-4

[24] Hinksman M, Naidu S, Loon K et al (2022) Long-term efficacy of endoscopic pilonidal sinus treatment: a single-centre Australian experience[J]. Anz j surg 92(5):1142–1148 35347830 10.1111/ans.17666

[25] Gulcu B, Ozturk E (2022) Endoscopic pilonidal sinus treatment vs. laser-assisted endoscopic pilonidal sinus treatment: short-term results from a retrospective case-matched study[J]. Tech coloproctol 26(4):271–277 35025023 10.1007/s10151-021-02568-8

[26] Manigrasso M, Velotti N, Sosa Fernandez LM et al (2021) Endoscopic Approach to Recurrent Pilonidal Sinus: A Retrospective Analysis[J]. J laparoendosc adv s 31(1):1–5 10.1089/lap.2020.0252 32678724

[27] Gulcu B, Ozturk E (2021) Endoscopic Pilonidal Sinus Treatment: Rapid Recovery, Satisfactory Success, and High Quality of Life[J]. Surg laparo endo per 31(6):711–715 10.1097/SLE.0000000000000974 34310558

[28] Foti N, Passannanti D, Libia A et al (2021) A minimally invasive approach to pilonidal disease with endoscopic pilonidal sinus treatment (EPSiT): a single-center case series with long-term results[J]. Tech coloproctol 25(9):1045–1054 34110535 10.1007/s10151-021-02477-w

[29] Dotlacil V, Rygl M, Frybova B (2021) Initial experience with minimally invasive treatment of pilonidal sinus in children[J]. Videosurgery miniinv 16(2):417–422 10.5114/wiitm.2020.100714 PMC8193749 34136040

[30] Meinero P, La Torre M, Lisi G et al (2019) Endoscopic pilonidal sinus treatment (EPSiT) in recurrent pilonidal disease: a prospective international multicenter study[J]. Int j colorectal dis 34(4):741–746 30719564 10.1007/s00384-019-03256-8

[31] Pini Prato A, Mazzola C, Mattioli G et al (2018) Preliminary report on endoscopic pilonidal sinus treatment in children: results of a multicentric series[J]. Pediatr surg int 34(6):687–692 29675752 10.1007/s00383-018-4262-0

[32] Pappas AF, Christodoulou DK (2018) A new minimally invasive treatment of pilonidal sinus disease with the use of a diode laser: a prospective large series of patients[J]. Colorectal dis 20(8):O207–o214 29878584 10.1111/codi.14285

[33] Georgiou GK (2018) Outpatient laser treatment of primary pilonidal disease: the PiLaT technique[J]. Tech coloproctol 22(10):773–778 30306277 10.1007/s10151-018-1863-5

[34] Dessily M, Charara F, Ralea S et al (2017) Pilonidal sinus destruction with a radial laser probe: technique and first Belgian experience[J]. Acta chir belg 117(3):164–168 28056720 10.1080/00015458.2016.1272285

[35] Meinero P, Stazi A, Carbone A et al (2016) Endoscopic pilonidal sinus treatment: a prospective multicentre trial[J]. Colorectal dis 18(5):O164–O170 26946340 10.1111/codi.13322

[36] Gülen M, Emral A (2025) Efficacy and Outcomes of Laser Treatment in Pilonidal Sinus Disease[J]. Ankara Eğitim ve Araştırma Hastanesi Tıp Dergisi 57(3):120–123

[37] Erculiani M, Mottadelli G, Carlini C et al (2022) Long-term results of EPSiT in children and adolescents: still the right way to go[J]. Pediatr surg int 38(9):1257–1261 35779104 10.1007/s00383-022-05162-7

[38] Gallo G, Goglia M, De Simone V et al (2025) Intra-operative ultrasound in the surgical treatment of complex and recurrent pilonidal disease: a retrospective, observational, single-center study[J]. Int j colorectal dis 40(1):203 40974390 10.1007/s00384-025-04961-3 PMC12450213

[39] Bilgin IA, Tanal M, Ramoglu N et al (2023) Short- and mid-term results of diode laser treatment in pilonidal sinus disease and the role of endoscopic camera use on outcomes[J]. Tech coloproctol 27(10):921–928 37356014 10.1007/s10151-023-02831-0

